# Longitudinal observation of solitary fibrous tumor translation into malignant pulmonary artery intimal sarcoma

**DOI:** 10.1186/s13019-020-01271-3

**Published:** 2020-09-01

**Authors:** Rui Luo, Yingshuo Jiang, Yue Huang, Xin Chen, Feng Wang

**Affiliations:** 1 Department of Nuclear Medicine, Nanjing First Hospital, Nanjing Medical University, 68th Changle Road, Nanjing, 210006 China; 2 Department of Cardiothoracic Surgery, Nanjing First Hospital, Nanjing Medical University, 68th Changle Road, Nanjing, 210006 China; 3 Department of Pathology, Nanjing First Hospital, Nanjing Medical University, 68th Changle Road, Nanjing, 210006 China

**Keywords:** Pulmonary artery intimal sarcoma, Solitary fibrous tumor, Malignant potential, ^18^F-FDG, Positron emission tomography

## Abstract

**Background:**

Pulmonary artery intimal sarcoma (PAIS) is a rare malignant tumor that was usually misdiagnosed as chronic pulmonary thromboembolism.

**Case presentation:**

We previously reported a solitary fibrous tumor in the pulmonary artery presented with acute pulmonary embolism, which was identified by ^99m^Tc-Galacto-RGD_2_ imaging. However, this patient had a recurrence in situ two-year after surgery, post-operative pathology revealed pulmonary artery intimal sarcoma. At one-year post-operation, ^18^F-FDG PET/CT was performed for exclusion of tumor metastasis, which showed FDG avid lesion in the T5, T10, and L5 vertebral bodies, as well as in bilateral ilium and right ischium.

**Conclusions:**

This is the first longitudinal observation of a solitary fibrous tumor (SFT) development into a pulmonary artery intimal sarcoma (PAIS) and presented with multiple bone metastases.

## Background

Pulmonary artery intimal sarcoma (PAIS) is a rare malignant tumor that may be misdiagnosed as chronic pulmonary thromboembolism [1, 2]. We previously reported a solitary fibrous tumor in the pulmonary artery presented with acute pulmonary embolism, which was identified by ^99m^Tc-Galacto-RGD_2_ imaging [3]. However, this patient had a recurrence in situ two-year after surgery, post-operative pathology revealed pulmonary artery intimal sarcoma. This is the first longitudinal observation of a solitary fibrous tumor (SFT) development into a pulmonary artery intimal sarcoma (PAIS) and presented with multiple bone metastases.

## Case presentation

**Fig. 1:**
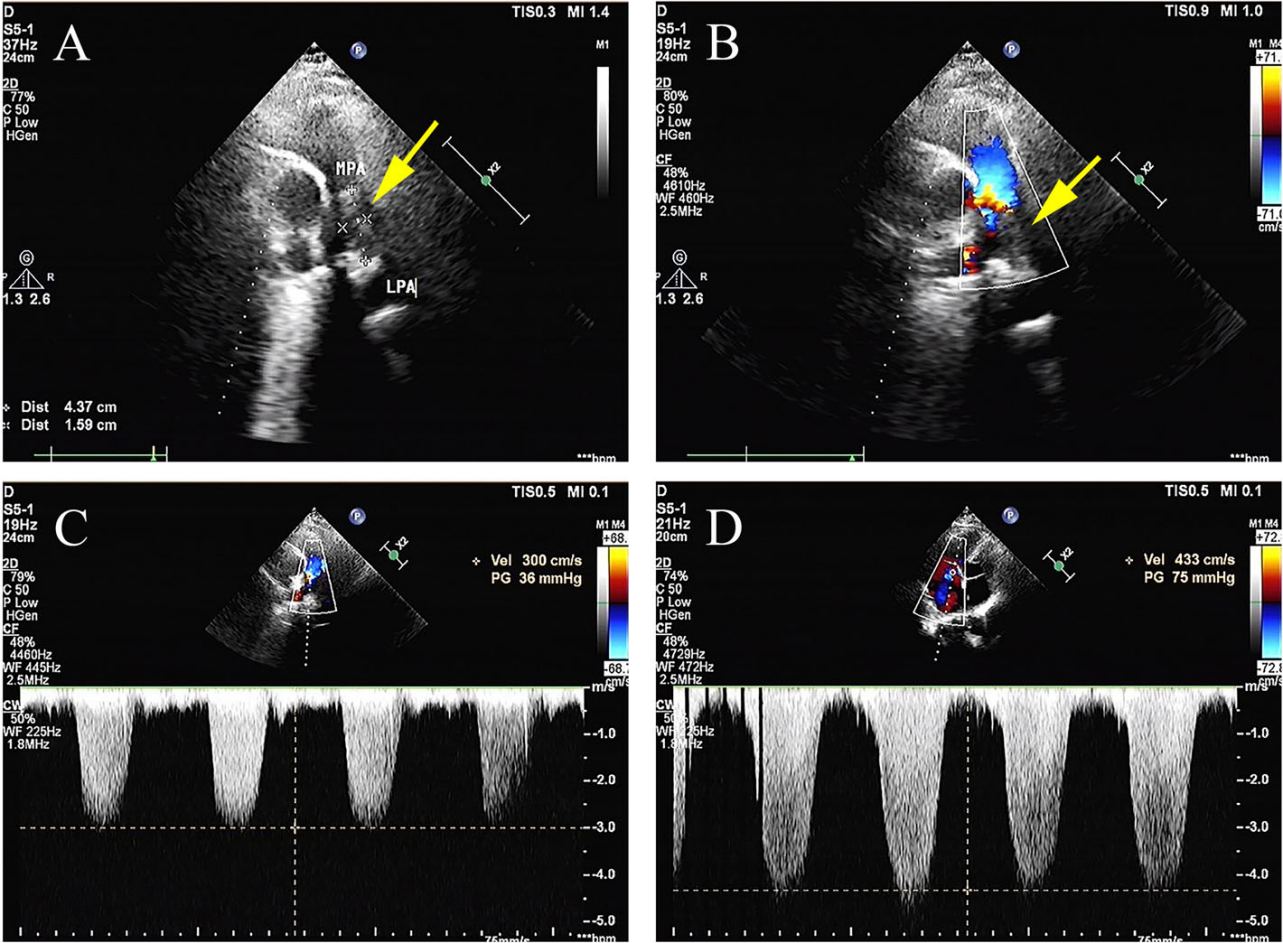
Evaluation of mass located in main pulmonary artery and bifurcation of pulmonary artery with echocardiography. **a**, **b** Transthoracic echocardiography: a 43.7 mm × 15.9 mm solid echo-level mass which attached to paries lateralis of the main and bifurcation of pulmonary artery. **c** Color Doppler. No significant blood flow was seen in the left pulmonary artery, whereas partial blood flow passed in right pulmonary artery. **d** Ultrasonic spectroscopy: the turbulent flow passed the mass

**Fig. 2:**
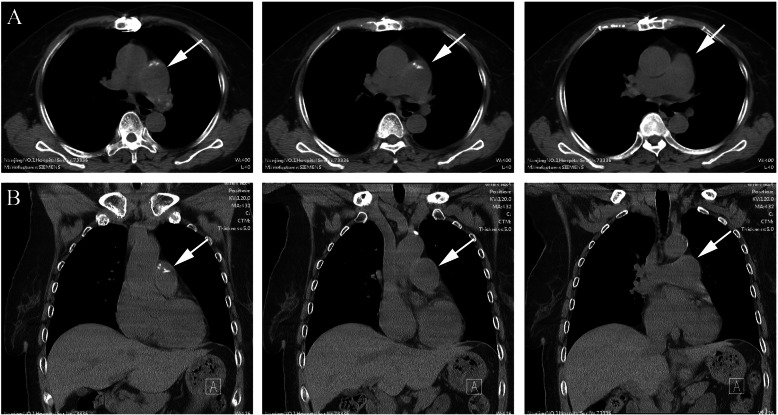
The chest CT showed partial high density shadow of pulmonary trunk and left branch, but no significant mass in the lung. **a** Transaxial, **b** Coronal, the lesion indicated by arrow

**Fig. 3:**
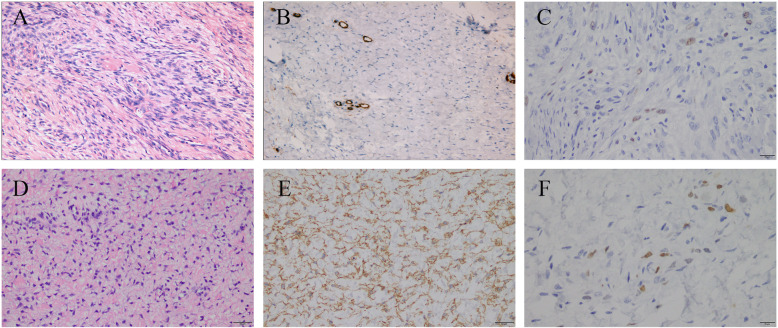
Pathological and immunochemical findings showed tumor progress and developed into PAIS. **a**, **b**, **c** First post-operative pathology **d**, **e**, **f** Second post-operative pathology. HE staining: (**a**, **d**) CD34 (**b**, **e**) Ki-67 (**c**, **f**)

**Fig. 4:**
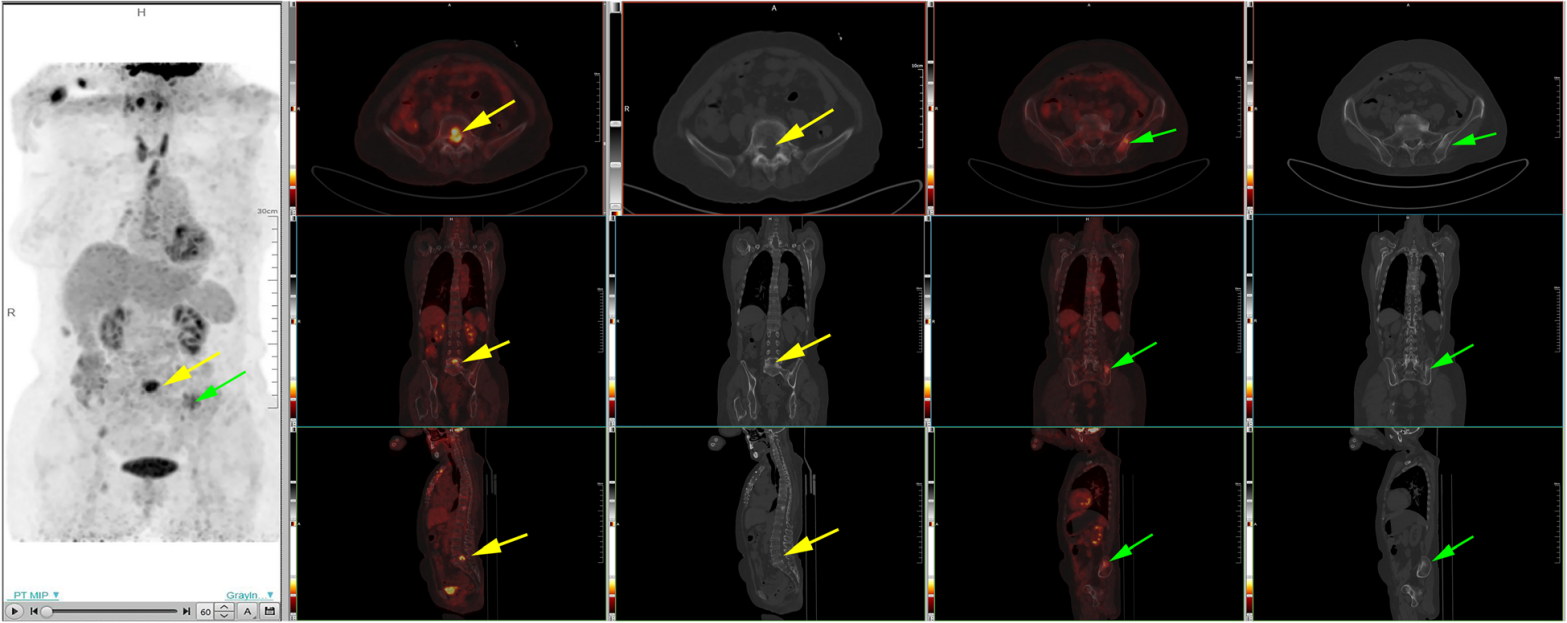
^18^F-FDG PET/CT showed multiple hypermetabolic lesions which was consistent with lytic bone changes in the T5, T10, and L5 vertebra, as well as bilateral ilium and right ischium

The 70-year-old female patient we previously reported with pulmonary solitary fibrous tumors (SFT), presented with increasingly aggravated oppression in chest but released after rest. Transthoracic echocardiography revealed a 43.7 mm × 15.9 mm solid echo-level mass which attached to the lower part of the main pulmonary artery and the beginning of left pulmonary artery, no significant blood stream was seen in the left pulmonary artery, whereas partial blood stream passed the right pulmonary artery. Ultrasonic spectroscopy found the velocity of blood flow of tricuspid valve was 4.3 m/s, which was turbulent flow (Fig. 1). Chest CT showed partial higher density shadow of pulmonary trunk and left branch (Fig. 2). Herein, after cardiopulmonary bypass was established, the aorta was opened and the mass was carefully resected. The surface of the mass was smooth, white and elastic and attached to the main pulmonary artery and bilateral pulmonary artery. The hematoxylin and eosin (H&E) staining showed the majority of cells were proliferating spindle cells with significant heterogeneity and interspersed vessel branching. Immunohistochemical analysis (ICH) showed: CD34(3+), CD99(−), SMA(−), d2–40(+), SOX10(−), NF(−) STAT6(±), CK(−), Ki-67(10%) S-100(−), compared with first post-operative surgery, spindle cell showed more atypia and higher expression of CD34 (Fig. 3). These pathological findings revealed that recurrent lesion was artery intimal sarcoma. At one-year post-operation, ^18^F-FDG PET/CT was performed for exclusion of tumor metastasis, which showed FDG avid lesion in the T5, T10, and L5 vertebral bodies, as well as in bilateral ilium and right ischium (Fig. 4).

## Discussions

PAIS grows within the lumen of pulmonary arteries and eventually occludes those vessels. Common symptoms of primary pulmonary arterial sarcoma include dyspnea, chest pain, edema, cough, and hemoptysis [4, 5], it mimics pulmonary embolism (PE), which is characterized by pulmonary artery luminal narrowing or occlusion in computer tomography (CT) [6]. How to diagnose the tumor originated from pulmonary artery accurately is great challenge in the clinical, because of insidious onset and symptoms indistinguishable from pulmonary thromboembolic diseases.

This rare case had relapse of chest oppression because of pulmonary artery intimal sarcoma, who was previously diagnosed as solitary fibrous tumor after the first surgery. Echocardiography reflected blood stream, calcification and blood velocity, which was useful for the detection of PAIS. ^18^F-FDG image showed multiple hyper-metabolic lesions consistent with lytic bone changes after the second surgery. Several case reports validated ^18^F-FDG PET/CT had some merits in the diagnosis of mesenchymal derived sarcomas,^18^F-FDG uptake in the higher-grade sarcoma correlated with mitotic count and grade [5–8]. The second post-operative pathology showed much more atypia and heterogeneity, significantly higher expression of CD34, which is hallmark of neo-vasculature, the proliferation index of Ki-67 increased as well.

This is the first report of longitudinal observation of SFT development into a PAIS with multiple bone metastases. Till now, the mechanism of tumor initiation, development and metastasis is not fully elucidated, the biological behavior is not fully identified, which is really great challenge in the oncology. Nuclear medicine and molecular imaging can describe tumor metabolism, receptor expression and angiogenesis, which serves as valuable surrogates of metastasis and prognosis. Integrin αvβ_3_ overexpressed on activated endothelial cells, and medicated tumor growth, local invasiveness and metastatic potential. In this case, if antitumor treatment was given after second operation, maybe the multiple metastasis can be avoided, which has been observed in the well differentiated neuroendocrine tumor. If patient was examined on time with ^99m^Tc-Galacto-RGD_2_ SPECT/CT and ^18^F-FDG PET/CT, metastatic lesions would be found earlier than morphological changes. ^99m^Tc-Galacto-RGD_2_ SPECT/CT was critical in the diagnosis of SFT, and further revealed metastatic potential and angiogenesis which led to development and metastasis. Therefore, we recommend that patient undergoes systemic evaluation of preoperative ^99m^Tc-Galacto-RGD_2_ SPECT/CT, and necessary nuclear medicine and molecular imaging after surgery to early detect the metastasis. However, whether to perform systematic treatment, it is worthy of further discussion. This longitudinal observation of SFT development and progression to malignant pulmonary artery intimal sarcoma, which sheds light on tumor development and metastasis.

## Conclusion

Intimal sarcoma of the pulmonary artery is a rare malignant tumor that may be developed from solitary fibrous tumor, which sheds light on the mechanism of tumor development and metastasis. ^99m^Tc-Galacto-RGD_2_ SPECT/CT and ^18^F-FDG PET/CT, may evaluate the biological behavior and prognosis. This case report addresses SFT development into a PAIS with multiple bone metastases.

## Data Availability

Not applicable.
